# Commentary: Efficacy of Follicle-Stimulating Hormone (FSH) Alone, FSH + Luteinizing Hormone, Human Menopausal Gonadotropin or FSH + Human Chorionic Gonadotropin on Assisted Reproductive Technology Outcomes in the “Personalized” Medicine Era: A Meta-analysis

**DOI:** 10.3389/fendo.2017.00264

**Published:** 2017-10-05

**Authors:** Johnny S. Younis

**Affiliations:** ^1^Reproductive Medicine, Department of Obstetrics and Gynecology, The Baruch Padeh Medical Center, Poriya, Tiberias, Israel; ^2^Faculty of Medicine in Galilee, Bar-Ilan University, Safed, Israel

**Keywords:** follicle-stimulating hormone, luteinizing hormone, GnRH analogues, LH threshold, clinical pregnancy

Santi et al. have performed a substantial meta-analysis to explore the benefit of LH activity preparations in addition to FSH in GnRH analogs cycles during controlled ovarian stimulation in the ART setting (1). The present meta-analysis targeted the general infertile population, placing no limits of age. Moreover, it is the first in which all gonadotropin combinations were considered. The author’s primary analysis targeted which LH activity supplemented: rLH, hMG, or hCG may have an effect on number of oocytes achieved. While, number of embryos, implantation, and live birth were considered as secondary end-points. Furthermore, sub-group analysis was made in accordance with the GnRH analog employed, agonist, or antagonist, respectively. The authors concluded that FSH alone results in higher oocyte number, while hMG improves the number of mature oocytes and embryos as well as increase implantation rate, whereas rLH supplementation improves pregnancy rate.

Although numerous previous meta-analyses has been already published exploring the added value of LH activity preparations to COH protocols, the topic continues to be actively debated and controversial, causing some confusion between practitioners. The present meta-analysis has a major advantage in accumulating large set of data targeting all LH activity preparations in clinical use. In doing so, the authors gathered all related controlled studies; however, randomization of the selected trials was not considered a strict inclusion criterion.

The results of the present meta-analysis differ from previous meta-analyses, specifically those targeting rLH supplementation. Earlier publications have made an effort to include only randomized controlled trials. Recombinant LH supplementation in the same setting was not found to improve pregnancy rate in the general infertile population (2–6). Furthermore, while previous meta-analyses have performed sub-group analysis to examine rLH supplementation on pregnancy rate in low responders and in women above 35 years of age, finding a value-added outcome, this was not attempted in current analysis (4, 7, 8). More important, no effort was made to examine the effect of LH activity preparations on hypo-responders, i.e., young normo-gonadotropic women who respond suboptimally to rFSH monotherapy following GnRH analog treatment. These add-ons would have been important guides to contemporary ART practice in the era of “personalized medicine.”

The sub-group analysis performed in the present investigation to distinguish between GnRH agonist and antagonist administration is notable. While LH activity preparations were shown to improve outcome measures when the GnRH agonist protocol was employed, this was not the case when LH activity was supplemented to the GnRH antagonist protocol. This has already been shown investigating pregnancy rate and focusing on rLH supplementation to GnRH antagonist cycles in the ART setting (2, 6).

Other potential confounders should be enlightened and taken into consideration when addressing this long-standing complex topic. It has been demonstrated that only 1% of LH receptors should be occupied to stimulate normal steroidogenesis in the ovulatory process (9). Residual endogenous LH level following the GnRH analog administration may be sufficient to achieve a competent oocyte ready for fertilization and implantation. In addition, LH and LH receptor (LHCGR) polymorphisms has been shown to affect ovarian response and predict pregnancy in normo-gonadotropic young infertile women. These polymorphisms may result in hypo-sensitivity to exogenous FSH during COH (10, 11). Furthermore, LH receptors have been identified in the human endometrium raising the possibility that LH supplementation may have a role in the implantation process and pregnancy achievement (12).

Taken together, the topic of LH activity supplementation to FSH therapy in the GnRH analog cycles during the ART setting is intricate and involves several factors that should be adequately addressed and analyzed. The population targeted, LH activity preparation employed and GnRH analog administered seems to have a diverse effect on clinical outcome measures and pregnancy achievement. Furthermore, other confounding factors are present that may be difficult to control for in randomized controlled trials and subsequent meta-analyses.

All told, due to the controversy available between different meta-analyses, it is still not clear today which groups of patients may benefit from LH activity supplementation to FSH treatment in GnRH analogs cycles. It is possible that the time has come to reach a consensus definition of LH threshold for adequate ovarian folliculogenesis and steroidogenesis in GnRH analog-treated cycles. An accurate definition of LH threshold in this setting may contribute to the discussion of which groups of women may benefit from LH activity supplementation.

## Author Contributions

The author confirms being the sole contributor of this work and approved it for publication.

## Conflict of Interest Statement

The author declares that the research was conducted in the absence of any commercial or financial relationships that could be construed as a potential conflict of interest.
